# Muscle spindles in the rhesus monkey platysma

**DOI:** 10.1111/joa.13604

**Published:** 2021-12-10

**Authors:** Christian Albrecht May, Kerstin Mätz‐Rensing, Daniel Aschoff, Silvia Bramke

**Affiliations:** ^1^ Department of Anatomy Medical Faculty Carl Gustav Carus, TU Dresden Dresden Germany; ^2^ Pathology Unit German Primate Center Leibniz Institute für Primate Research Göttingen Germany

**Keywords:** cheek pouch, muscle spindle, platysma, rhesus monkey, striated muscle

## Abstract

The platysma of the rhesus monkey consists of two parts: a platysma myoides located similar to the human platysma, and a platysma cervicale passing the dorsal cervical region and being in contact with the cheek pouch. Our investigation showed that the muscle fiber morphology was comparable in both parts. Muscle spindles were only present in regions connected to the cheek pouch and contained only nuclear chain fibers. It is tempting to speculate that they sense the filling of the cheek pouch rather than mimic activities.

## Graphical abstract

Macroscopic appearance of a left in situ (A) and extracted (B) platysma (7‐year‐old male rhesus monkey): note the compact platysma cervicale (double‐headed arrow) covering partly the cheek pouch (asterisk). The platysma myoides (white arrowheads) forms single muscle bundles separated by connective tissue strains. Single pure‐nuclear‐chain fiber muscle spindles (C) were in close association to the cheek pouch.

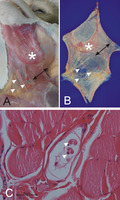

## 1 INTRODUCTION

Although the mimetic muscles are usually referred to as containing no corpuscular sensors, we recently described numerous muscle spindles in the human platysma (May et al., 2018). In rhesus monkeys (*Macaca* (*M*.) *mulatta*), the platysma is present but shows a distinctly different location if compared to the human: “The platysma muscle in *M*. *mulatta* is similar to many other primates in being relatively flat, thin and expansive with fibers passing through the dorsal cervical region, inferior to the pinna, splitting around the cheek pouch and attaching into the inferolateral edge of the zygomaticus major and levator anguli oris muscles” (Burrows et al., 2009, p. 323). This type of platysma is named platysma cervicale, in contrast to the human platysma myoides. While the platysma cervicale is present in numerous primates including gibbons (Burrows et al., 2011) and orang utans (Boyle et al., 2020), it is virtually absent in chimpanzees and gorillas and replaced by the myoides type (Boyle et al., 2020; Diogo et al., 2013). Still, some muscle bundles represent the platysma myoides in all primates. In the human, occasionally a remnant of the platysma cervicale appears as a transversus nuchae muscle (Boyle et al., 2020).

The aim of the present research was to investigate the microscopic appearance of the whole rhesus monkey platysma with a special focus on corpuscular sensors.

## 2 MATERIAL AND METHODS

### 2.1 Tissue preparation

Platysma specimens were provided from five adult rhesus monkeys (three females aged 14, 16 and 20 years, two males aged 7 and 22 years). The animals were inbreeds from the German Primate Center Göttingen and sacrificed due to other protocols. The platysma cervicale and platysma myoides were thoroughly exposed, taken out as a whole, and immersion fixed in 4% formaldehyde. After shipping to Dresden, the specimens were cut into pieces, dehydrated in an ascending series of ethanol, and embedded in paraffin.

### 2.2 Histology

Serial sections (5‐ to 10‐μm thick) of each specimen were performed in different planes and selected sections were stained with hematoxylin and eosin (H&E). The sections were examined on a Zeiss Jenamed2 microscope (Carl Zeiss AG) and images were recorded by using a Digital Sight DS‐Fi1 camera (Nikon AG).

The muscle fiber diameters were measured in two sections of each muscle specimen (5 animals × 4 sections). In each section, 100 neighboring muscle fibers were measured in a random region. The visual appearance of all muscles was homogenous with no signs of regional differences. The thickness of the muscle was measured in cross‐sectional planes: for that purpose, the area covered by muscle fibers of a standardized length (500 µm) was measured and a mean thickness was determined by dividing the area through the length. This procedure was performed in five different regions of each specimen.

## 3 RESULTS

**FIGURE 1:**
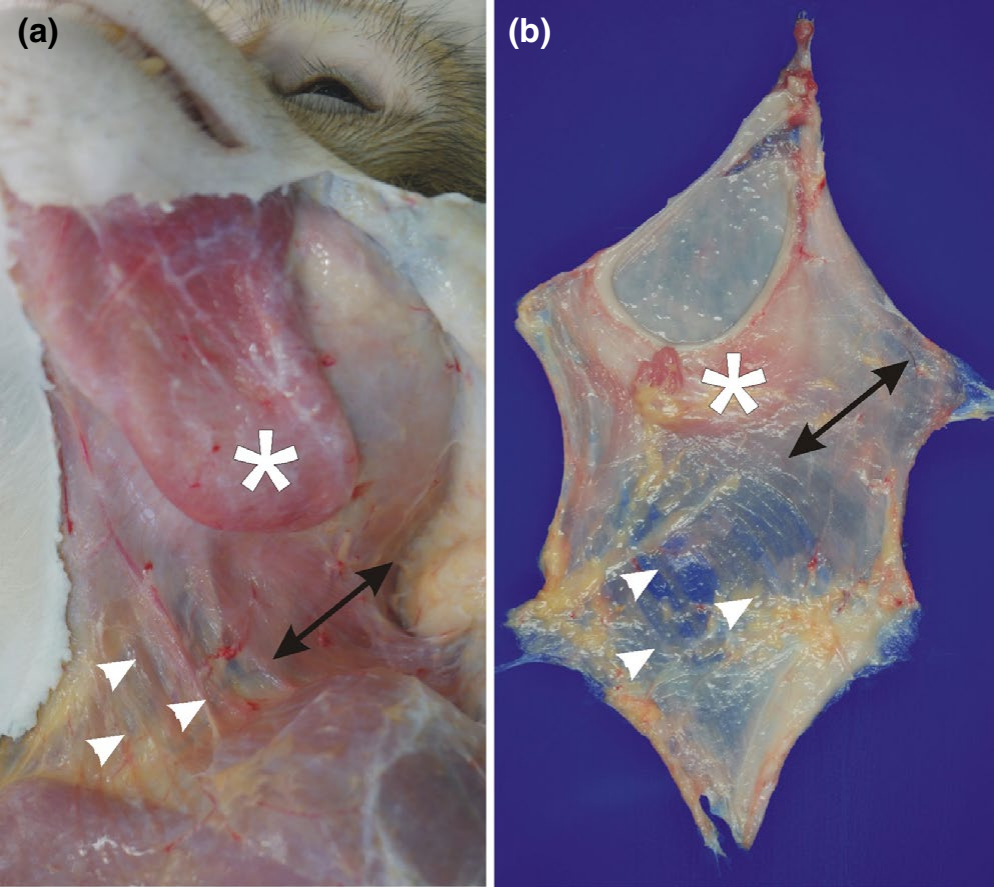
Macroscopic appearance of a left in situ (a) and extracted (b) platysma (7‐year‐old male rhesus monkey): note the compact platysma cervicale (double‐headed arrow) covering partly the cheek pouch (asterisk). The platysma myoides (white arrowheads) forms single muscle bundles separated by connective tissue strands

The rhesus monkey platysma could be divided into two parts (Figure 1): The more laterally located platysma cervicale was a flat solid muscle running from the dorsal cervical region toward the region lateral to the orbicularis oris muscle covering partly the cheek pouch region. The medial located platysma myoides, in contrast, consisted of several muscle fiber bundles separated by connective tissue. They ran from the inferior ventrolateral cervical region toward the mental region.

### 3.1 Platysma cervicale (Figure 2)

**FIGURE 2:**
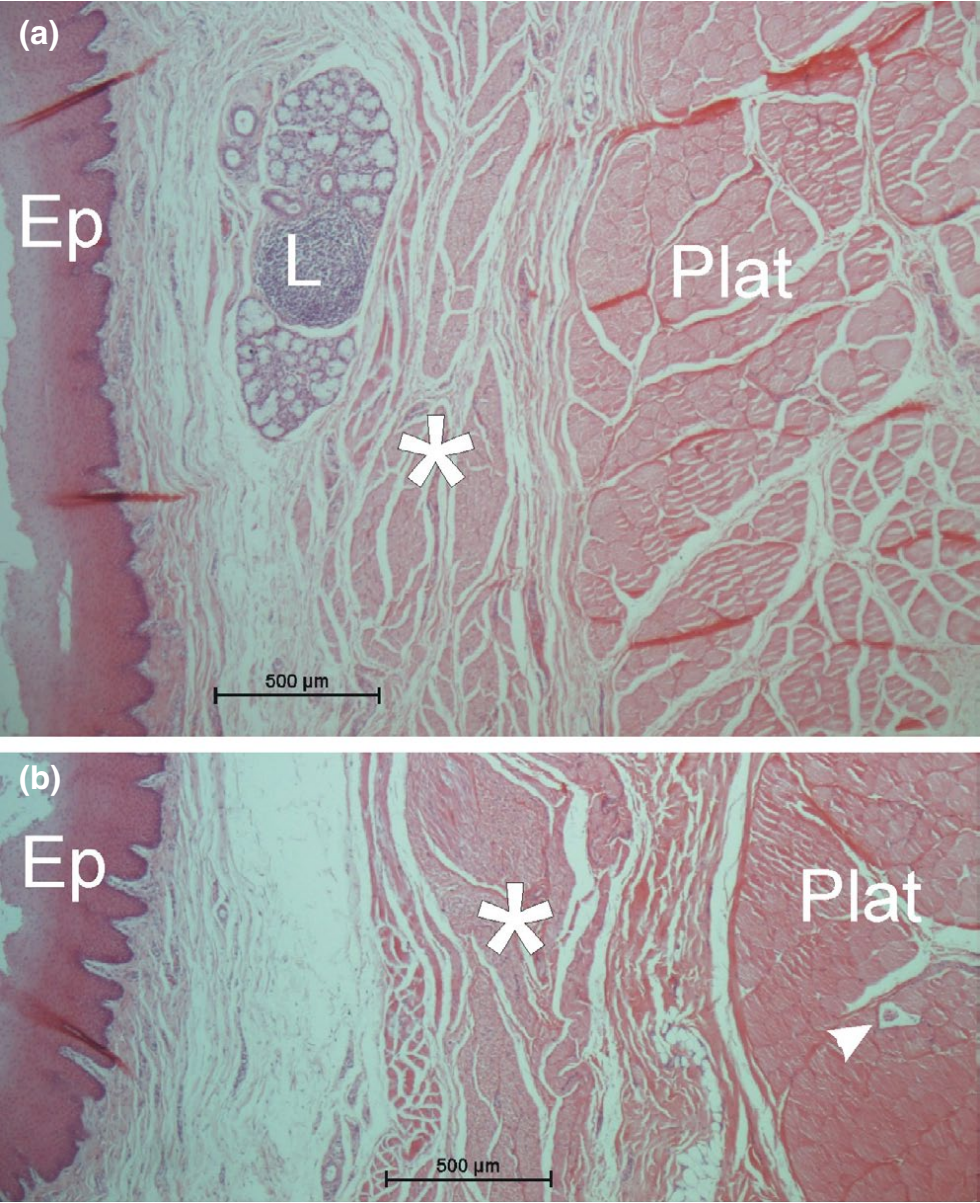
Microscopic appearance of the platysma cervicale (Plat) and the epithelium (Ep) and genuine muscle (asterisks) of the cheek pouch (HE stain). (a) Note a lymph follicle (L) and next to it mucous glands in the lamina propria between the epithelium and the cheek pouch muscle. (b) The variability in muscle fiber orientation of the cheek pouch muscle (asterisks) is clearly seen. The arrowhead marks a muscle spindle within the platysma cervicale

The muscle fibers of the platysma cervicale were well developed and showed single fiber diameters between 60 and 100 µm (mean diameter 86 ± 9 µm). The fibers were covered by a very thin endomysium and only a few perimysial septa. The thickness of the whole muscle was between 1.0 and 2.2 mm (mean 1.7 ± 0.3 mm). On neither side of the muscle, a continuous epimysium was present (Figure 2). The muscle lay within a loose connective tissue.

At the level of the cheek pouch, there was a second layer of striated muscle fibers between the platysma cervicale and the squamous epithelium (Figure 2). This muscle layer consisted of much smaller muscle fibers (diameter between 28 and 50 µm; mean diameter 37 ± 8 µm) and showed a distinctly different orientation of the fibers in different planes.

### 3.2 Platysma myoides (Figure 3)

**FIGURE 3:**
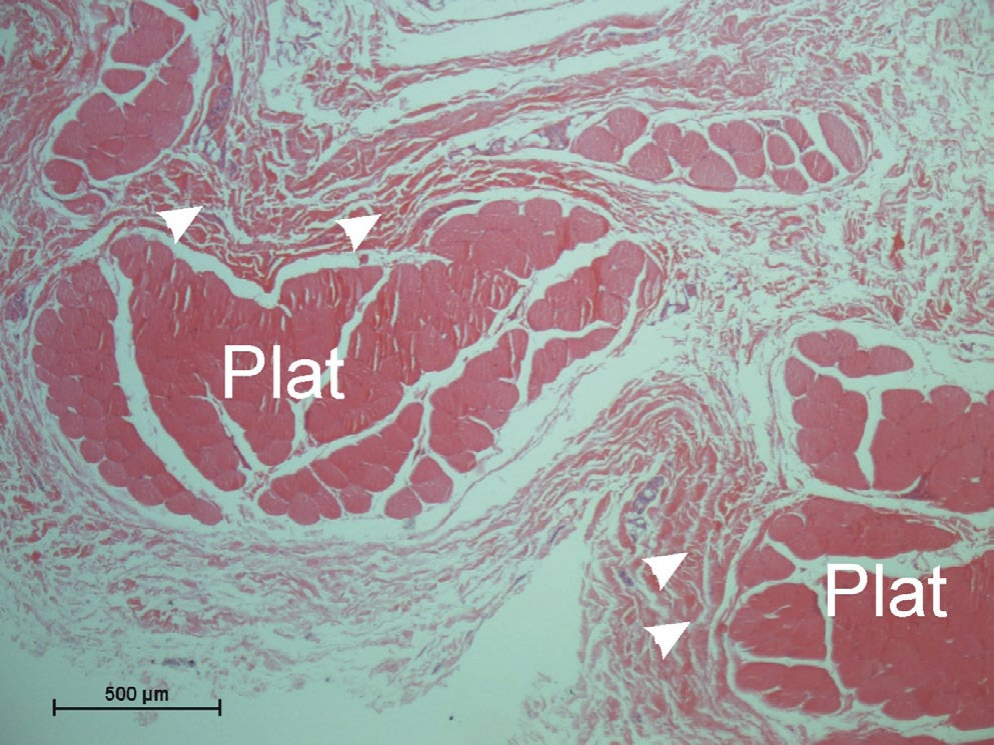
Microscopic appearance of the platysma myoides (HE stain). Note single muscle fiber bundles (Plat) separated by connective tissue. At places, the connective tissue shows densifications (arrowheads) without forming a continuous epimysium

The muscle fibers of the platysma myoides were comparable to the platysma cervicale concerning fiber diameter (mean diameter 85 ± 9 µm), muscular connective tissue arrangement, and muscle thickness (0.5–2.6 mm; mean 1.6 ± 0.5 mm). Between the individual bundles of muscle fibers, there was loosely arranged connective tissue and only occasional densified connective tissue as known for fascial structures could be observed toward the inner (profound) aspect of the muscle (Figure 3).

### 3.3 Corpuscular sensors and innervation

While most sections through the rhesus monkey platysma revealed solely extrafusal muscle fibers, five muscle spindles were observed in one completely sectioned platysma. These muscle spindles were projected all toward the cheek pouch. Four of the spindles were located in the platysma cervicale, one single spindle was located at the cranio‐lateral rim of the platysma myoides. Most of the individual muscle bundles of the platysma myoides were therefore without spindle sensors.

**FIGURE 4:**
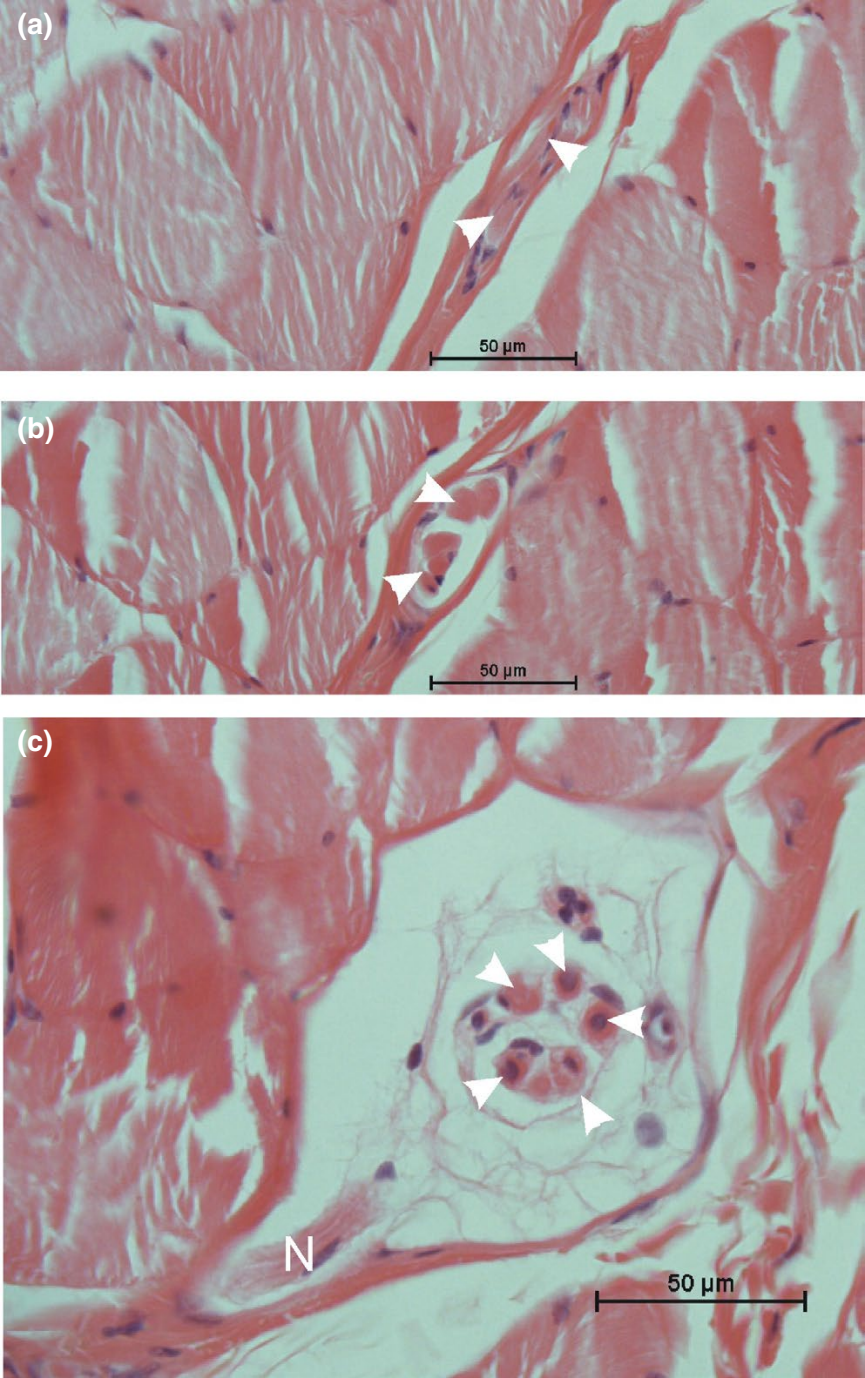
Microscopic appearance of a muscle spindle within the platysma cervicalis (HE stain). (a) At the termination of the muscle spindle, only two intrafusal muscle fibers (arrowheads) can be detected. (b) At the polar zone the spindle contains five intrafusal muscle fibers (arrowheads); the surrounding connective tissue sheath is tenuous. (c) At the equatorial zone, only single nuclei can be seen within the intrafusal muscle fibers (arrowheads) characterizing all fibers as nuclear chain fibers. N = entering nerve fibers

The muscle spindles contained only a thin connective tissue sheath (Figure 4). Between four and six small intrafusal muscle fibers were observed within this sheath. Due to their morphology, all fibers were classified as being nuclear chain fibers. Nuclear bag fibers were not present.

Besides the few muscle spindles, no other corpuscular sensors (Pacini corpuscles, Ruffini corpuscles) could be detected in any of the sections.

## 4 DISCUSSION

In contrast to the human platysma, where numerous muscle spindles were evenly distributed in the cranial part (May et al., 2018), the rhesus monkey platysma showed only few muscle spindles. These spindles showed a tenuous sheath, but otherwise all characteristic features (intrafusal muscle fibers, periaxial space, capillaries, innervation). Muscle spindles were also described in other facial muscles of the rhesus monkey (Lovell et al., 1977). The spindles in the rhesus monkey platysma contained only nuclear chain fibers pointing to a static registration rather than dynamic changes. In this respect, the close relation to the cheek pouch seems reasonable for an explanation.

Many monkey species develop a cheek pouch which serves not only for temporary food storage (Lambert, 2005; Smith et al., 2008) but also for social interaction (Hayes et al., 1992). The morphology of the monkey cheek pouch is only poorly described. The general layers are comparable to rodents and hamster (Ghoshal & Bal, 1990; Ryan, 1986), whose cheek pouches are mainly used for vascular and oncologic studies (Shubik, 1982; Svensjö, 1990). However, in contrast to the histomorphology of the hamster (Ghoshal & Bal, 1990), in the rhesus monkey, the lamina propria of the cheek pouch contained lymphatic tissue and mucous glands. The immune privilege suggested for the hamster cheek pouch (Arruda & Montenegro, 1995) might therefore not apply to the rhesus monkey. A genuine cheek pouch muscle is not mentioned in the list of facial muscles of the rhesus monkey (Burrows et al., 2009) but histology revealed its existence and its distinct difference to the overlaying platysma. This muscle is described in rodents and its innervation is related not only to the facial nerve but to branches from cervical nerves (Kawashima et al., 2020). It is tempting to speculate that the afferent nerve fibers of the platysma muscle spindles in the rhesus monkey are also more related to the cervical branches than to the facial nerve.

Since the genuine cheek pouch muscle fibers are somewhat interwoven it might be easier to measure changes in cheek pouch filling by the parallel platysma muscle fibers which are in close contact with the cheek pouch. This might explain the lack of muscle spindles in the genuine cheek pouch muscle and the distinct presence of muscle spindles in the platysma of the rhesus monkey. Unfortunately, there is no information about morphological defined functional circuits of the cheek pouch, its muscles, and its innervation. Functional studies did not include this specific region (Waller et al., 2008). More studies are necessary to define the role of the platysma in this respect.

## AUTHORS’ CONTRIBUTIONS

KMR and DA took tissue samples and reviewed the manuscript, CAM performed the evaluation and prepared the manuscript, and SB performed the staining and reviewed the manuscript.

## Figures and Tables

**FIGURE A1:**
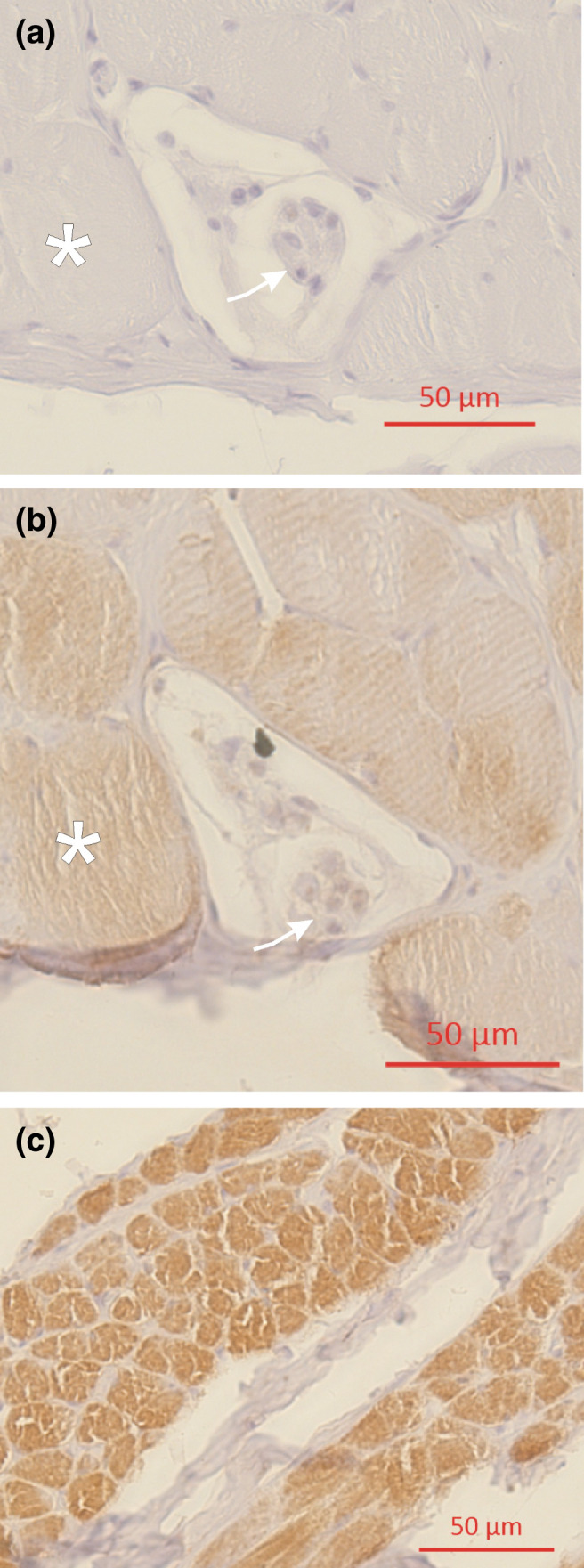
Immunohistochemical staining of monkey tissue with two human antibodies: S46 (a) and A4.74 (b and c). (a) There was no staining of the extrafusal (asterisks) and intrafusal (arrow) platysma muscle fibers. (b) There was some background, but no specific staining in most of the extrafusal (asterisks) and intrafusal (arrow) platysma muscle fibers. Single extrafusal muscle fibers of the platysma stained positive (data not shown). (c) All small muscle fibers of the cheek pouch muscle stained positive defining them possibly as type 2a/2x fibers.

## 1 METHODS

Immunohistochemistry was performed on selected sections of the rhesus monkey platysma using two human antibodies established in intrafusal fiber typing: the antibody S46 is an established antibody that identifies nuclear bag fibers. It is specific for the myosin heavy chain isoform “slow tonic” (MyHC‐slow tonic) which occurs in avian slow tonic muscle fibers, but in humans it appears only in intrafusal nuclear bag fibers or extraocular muscles (Schiaffino & Reggiani, 2011; Peikert & May, 2015; Sokoloff et al., 2007). The antibody A4.74 stains extrafusal type 2a/2x fibers and intrafusal nuclear chain fibers respectively (Liu et al., 2003; Österlund et al., 2013; Peikert & May, 2015). No data exists about specificity for both antibodies concerning the rhesus monkey. The presented data is therefore speculative and has to be discussed with caution. It is therefore not included into the main body.

## 2 RESULTS AND DISCUSSION

The staining with the antibody S46 was completely negative (Figure A1a). This might indicate no nuclear bag fibers in the muscle spindles of the rhesus monkey platysma. However, unfortunately we did not have a positive control. Therefore, we cannot prove that the antibody stains nuclear bag fibers in the rhesus monkey at all.

The antibody A4.74 showed an irregular staining pattern of the extrafusal muscle fibers of the platysma with most of the fibers being negative (Figure A1b). In contrast, all muscle fibers of the cheek pouch muscle were intensely positive stained (Figure A1c) indicating a homogenous group of type 2a/2x muscle fibers in this muscle. Since this antibody was also used for rat tissue (Smerdu & Soukup, 2008), its extrafusal staining pattern in the rhesus monkey might indeed represent the fiber typing indicated. Unfortunately, the intrafusal muscle fibers of the rhesus monkey platysma showed no specific positive staining reaction (Figure A1b). We therefore can only use morphological criteria to identify the intrafusal muscle fibers in the rhesus monkey platysma as being nuclear chain fibers.
